# Abdominal imaging findings in adult patients with Fontan circulation

**DOI:** 10.1007/s13244-018-0609-2

**Published:** 2018-04-05

**Authors:** Tae-Hyung Kim, Hyun Kyung Yang, Hyun-Jung Jang, Shi-Joon Yoo, Korosh Khalili, Tae Kyoung Kim

**Affiliations:** 1 Department of Radiology, Seoul National University Hospital, 101 Daehak-ro, Jongno-gu, Seoul, 03080 South Korea; 2 Department of Radiology, Seoul National University College of Medicine, 103 Daehak-ro, Jongnogu, Seoul, 03080 South Korea; 3 Department of Medical Imaging, Toronto General Hospital, 585 University Avenue, Toronto, ON M5G 2N2 Canada; 4 Department of Diagnostic Imaging, Hospital for Sick Children, 555 University Avenue, Toronto, ON M5J2L4 Canada

**Keywords:** Liver cirrhosis, Focal nodular hyperplasia, Heart defects, congenital, Diagnostic imaging, Digestive system neoplasms

## Abstract

**Abstract:**

The Fontan procedures, designed to treat paediatric patients with functional single ventricles, have markedly improved the patient’s survival into adulthood. The physiology of the Fontan circuit inevitably increases systemic venous pressure, which may lead to multi-system organ failure in the long-term follow-up. Fontan-associated liver disease (FALD) can progress to liver cirrhosis with signs of portal hypertension. Focal nodular hyperplasia-like nodules commonly develop in FALD. Imaging surveillance is often performed to monitor the progression of FALD and to detect hepatocellular carcinoma, which infrequently develops in FALD. Other abdominal abnormalities in post-Fontan patients include protein losing enteropathy and pheochromocytoma/paraganglioma. Given that these abdominal abnormalities are critical for patient management, it is important for radiologists to become familiar with the abdominal abnormalities that are common in post-Fontan patients on cross-sectional imaging.

**Teaching points:**

*• Fontan procedure for functional single ventricle has improved patient survival into adulthood.*

*• Radiologists should be familiar with unique imaging findings of Fontan-associated liver disease.*

*• Focal nodular hyperplasia-like nodules commonly develop in Fontan-associated liver disease.*

*• Hepatocellular carcinoma, protein-losing enteropathy, pheochromocytoma/paraganglioma may develop.*

**Electronic supplementary material:**

The online version of this article (10.1007/s13244-018-0609-2) contains supplementary material, which is available to authorized users.

## Introduction

The Fontan procedure was first introduced for patients with tricuspid atresia to allow systemic blood return to enter the pulmonary arteries without passage through a ventricle in 1968 by Fontan and Baudet [1, 2]. Subsequently, the procedure has been applied for most forms of functional single ventricles [3–6]. Surgical modifications of the procedure along with advances in postoperative management led to prolonged patient survival, frequently into adulthood [7–9].

The Fontan circuit allows the entire systemic venous return to enter the pulmonary vascular bed directly, bypassing the right ventricle. There are two ways to connect the systemic venous return to pulmonary arteries: either through the right atrium (namely atriopulmonary circulation) or by direct connection of superior vena cava (SVC) and inferior vena cava (IVC) to pulmonary arteries (namely cavopulmonary circulation). Cavopulmonary circulation is subdivided according to the type of IVC connection to the pulmonary artery either by intra-atrial lateral tunnel conduit or external cardiac conduit [10]. Due to the absence of a pumping ventricle, the transmission of pulmonary vascular resistance to the systemic venous circulation inevitably leads to chronically elevated systemic venous pressure. Thus, all organ systems of the body are exposed to chronic venous congestion, potentially leading to multi-system organ failure [11–13].

Fontan-associated liver disease (FALD) has been recognised as the disease process of liver structure and function resulting from the Fontan circulation, excluding other plausible causes such as viral hepatitis or alcohol toxicity. FALD is a broad term encompassing hepatic parenchymal change, hypervascular regenerative hepatic nodules and ultimately hepatic cirrhosis [14] . FALD is considered one of the major non-cardiac determinants of mortality in adult Fontan patients [15–18]. Other abdominal abnormalities found in adult patients with Fontan circulation include signs of portal hypertension such as ascites or varices, protein losing enteropathy (PLE) and pheochromocytoma/paraganglioma.

Although the imaging features of abdominal abnormalities after Fontan operation have been described in several publications mostly for the paediatric population [19–22], abdominal imaging findings in adult patients with Fontan circulation have not been well described in detail. In this review, we summarise the cross-sectional imaging findings of the hepatic and extrahepatic abnormalities in the abdomen in adult patients with Fontan circulation.

## Fontan-associated liver disease

Among the gastrointestinal system, the liver is particularly vulnerable to the elevated systemic venous pressure in Fontan circulation compared to other organ systems for four principal reasons [13, 14]. Firstly, most venous return from the gastrointestinal system passes through the liver. Secondly, portal venous flow to the liver relies on the pressure gradient between the hepatic and portal veins that decreases as the systemic venous pressure increases, in contrast to other organs in which the blood flow relies on the pressure gradient between the arterial and venous systems. Third, the natural response of diversion of gastrointestinal blood to other organs such as brain, heart and muscles further compromises already precarious circulation of the liver. Lastly, lymphatic drainage disturbance due to increased central venous pressure causes lymphatic-mediated hepatic congestion and sinusoidal dilatation, which in turn attributes to the liver injury [23].

Adult patients with Fontan circulation are prone to developing FALD as they age. It frequently results in chronic hepatic congestion, liver cirrhosis, portal hypertension, focal nodular hyperplasia (FNH)-like nodules and even hepatocellular carcinoma (HCC) [15, 24–26]. The possible mechanisms of hepatic dysfunction in FALD include chronic passive venous congestion, reversed blood flow with deep intrahepatic reflux during atrial contraction [11], and reduced hepatic blood supply with subsequent hypoxic damage from relatively low cardiac output after Fontan operation [27, 28]. Histopathological changes begin with sinusoidal dilatation, parenchymal atrophy and progressive sinusoidal fibrosis in the perivenular distribution, similar to that of right heart failure, but more exaggerated over time in patients with Fontan circulation [11].

The severity of FALD is positively related to the duration of the Fontan circulation, elevated hepatic venous pressure, failing Fontan circulation and increased serological markers such as hyaluronic acid or gamma-glutamyltransferase [11, 29, 30]. Monitoring FALD with imaging surveillance is essential in guiding patient management such as medical therapy for liver cirrhosis, placement on the liver transplantation list or treating HCC when it is detected. In post-Fontan patients who are considered for heart transplantation, severe FALD is considered a contraindication for heart transplantation or combined heart-liver transplantation [16, 26, 31].

Given the clinical importance of FALD in adult patients with Fontan circulation, various non-invasive screening/surveillance protocols for FALD have been suggested regarding the timing, intervals, and modalities [16, 18, 26, 32, 33]. Liver biopsy still remains the “gold standard” for assessing the severity of liver fibrosis and cirrhosis. However, reported liver changes secondary to FALD are not uniformly distributed and therefore liver biopsy may overestimate or underestimate the presence or nature of FALD [19, 28, 34]. Further, the risk of biopsy-related bleeding complication is likely to be higher due to the frequent use of warfarin and elevated systemic venous pressures [32, 35]. Therefore, non-invasive imaging surveillance may play a major role for the surveillance of FALD and its related changes.

### Imaging of hepatic parenchymal changes

**Fig. 1:**
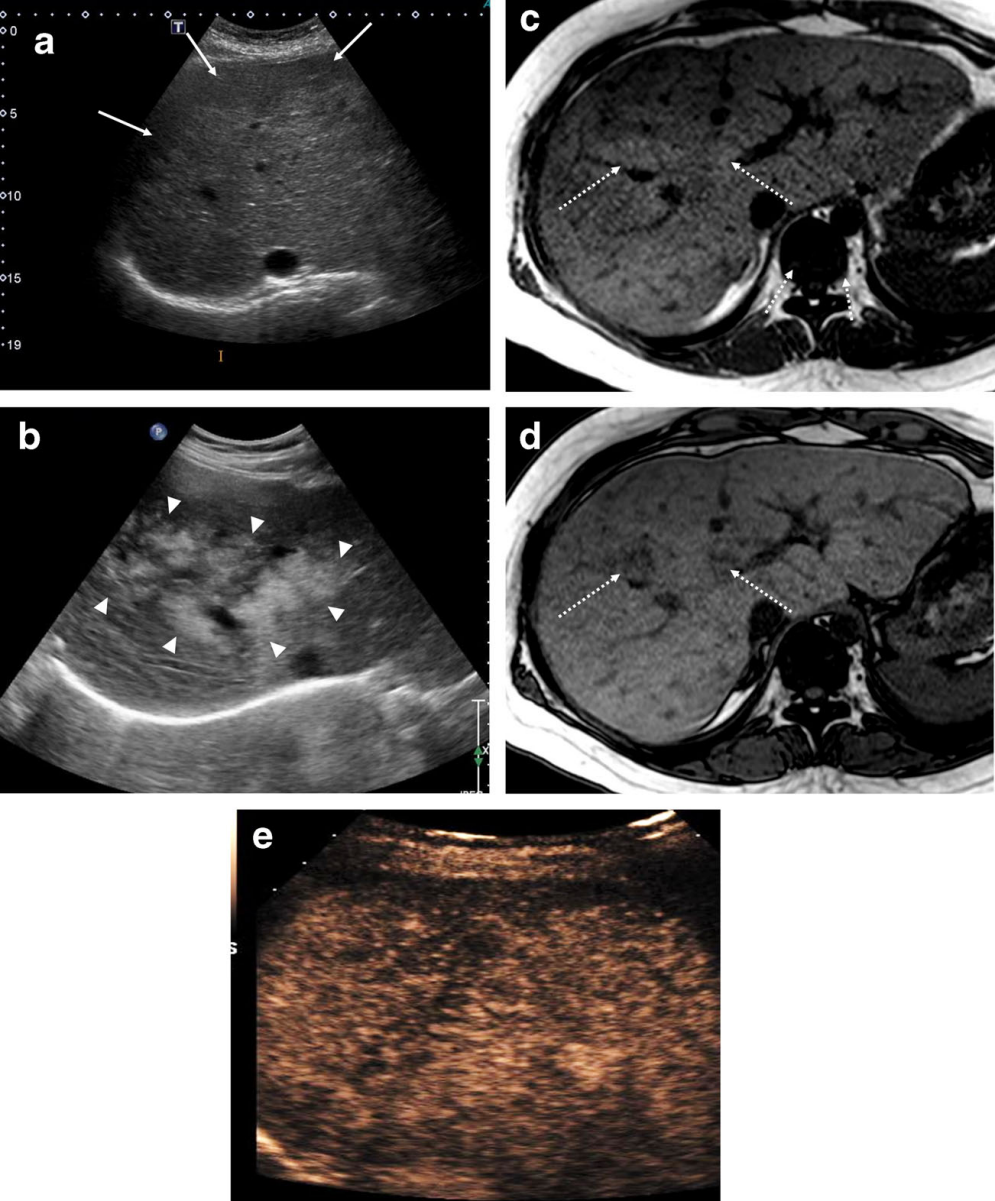
Hepatic parenchymal change in US in post-Fontan patients. **a** A 33-year-old woman with previous pulmonary atresia. US reveals heterogeneous parenchymal echotexture with central zone hyper-echogenicity and peripheral zone hypo-echogenicity (*arrows*). **b**-**d** A 30-year-old woman with previous hypoplastic right ventricle syndrome. **b** US shows irregular homogeneous hyperechoic mass-like areas (*arrowheads*) surrounding portal vein branches with geographic margin. **c**, **d** In-phase and opposed-phase axial T1-weighted MRI demonstrates increased signal intensity (SI) on in-phase image (**c**) and dropped SI on opposed-phase image (**d**), suggesting fat deposit (*dotted arrows*). These areas correspond to the hyperechoic mass-like areas on US. **e** A 48-year-old woman with previous double outlet right ventricle. Contrast-enhanced ultrasound image obtained at 3 min shows marked coarse and heterogeneously weak delayed parenchymal enhancement

The liver may appear normal or slightly hypoechoic on ultrasound (US) at the early stage of congestive hepatopathy. As the fibrosis develops, coarse heterogeneous hyperechoic parenchymal pattern and surface nodularity are visualised [30] (Fig. 1a). The liver is often enlarged with caudate lobe hypertrophy similar to Budd-Chiari syndrome. Correlations have been reported between the severity of US features and the degree of hepatic fibrosis or cirrhosis [20, 36, 37]. Irregular parenchymal fatty infiltration with perivascular distribution can be seen in US and in magnetic resonance imaging (MRI) with in-phase and out-of-phase sequences (Fig. 1b-d) [22, 38]. On MRI, heterogeneous hyperintensity on T2-weighted images and hypointensity on T1-weighted images in the periphery of the liver, which is commonly seen in acute or subacute Budd-Chiari syndrome, is not seen in FALD [39–41], probably due to chronic gradual congestive changes of the liver over a long period. Diffusion-weighted imaging (DWI) can help estimating the degree of hepatic fibrosis. Wolff et al. [21] reported that low apparent diffusion coefficient values in DWI may reflect progressive liver damage due to chronic congestion and potential hypoperfusion in post-Fontan patients.

**Fig. 2:**
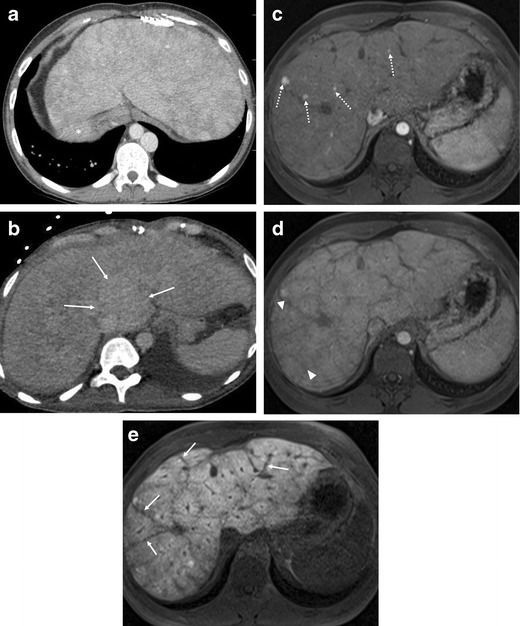
Hepatic parenchymal change in CT and MRI in post-Fontan patients. **a** A 22-year-old woman with previous right atrial isomerism and pulmonary atresia. Axial CT image in portal venous phase demonstrates reticular regions of poor parenchymal enhancement in the periphery of the liver, which are prominent in the portal venous phase. Note that the stomach is located on the right side (*asterisk*) with left-sided liver and inferior vena cava (heterotoxy syndrome). **b** A 36-year-old woman with previous hypoplastic right ventricle syndrome. Axial CT image in portal venous phase demonstrates diffuse ill-defined inhomogeneous enhancement in the liver. Note that the caudate lobe hypertrophy with relative homogeneous attenuation (*arrows*). **c**-**e** A 22-year-old woman with previous hypoplastic right ventricle syndrome. Axial contrast-enhanced T1-weighted MR images with gadoxetate disodium–enhanced MR images obtained in the arterial phase (**c**), transitional phase (**d**) and hepatobiliary phase (**e**). **c** Multiple small arterial-enhancing foci (*dotted arrows*) with contrast agent retention in the hepatobiliary phase are scattered in the liver, in keeping with focal nodular hyperplasia-like nodules. **d** The arterial-enhancing foci show no delayed washout. Note that heterogeneous parenchymal enhancement is prominent in the right posterior segment of the liver (*arrowheads*). **e** The liver shows heterogeneously mildly decreased contrast material uptake, possibly reflecting decreased hepatic function and congestion. Also, low-signal intensity reticular bands with no contrast material retention (*arrows*) which may be due to dilated veins / fibrous septa and tend to spare regions around the portal triads while coming into direct contact with hepatic veins

In addition to the morphological changes of the liver, markedly heterogeneous hepatic enhancement with mosaic or reticular patterns, resulting from relatively slow and poor enhancement near the congested hepatic veins, is the most common imaging feature in FALD [19, 38]. Contrast-enhanced ultrasound (CEUS) in our anecdotal experience shows heterogeneous and decreased enhancement of the liver in the portal venous phase similar to cirrhotic liver of other common aetiologies (Fig. 1e). The abnormal enhancement is more prominent in the periphery of the liver than the central portion and is most evident in the portal venous phase (Fig. 2a-d). The hypertrophic caudate lobe often shows more homogeneous enhancement compared to the rest of the liver [13] (Fig. 2b). Hepatobiliary phase MRI using a hepatocyte-specific contrast agent (gadoxetate disodium; Primovist; Bayer, Berlin, Germany) often shows heterogeneous hypoenhancement in the hepatobiliary phase, which reflects decreased hepatic function [42] (Fig. 2e). Hepatic fibrosis may be seen as reticular hyperenhancement in the delayed phase of CT or MRI. It has been reported that the degree of hepatic dysfunction is not well correlated with the degree of fibrosis in post-Fontan patients [18, 43]. Therefore, follow-up with clinical and laboratory tests is also essential.

### Imaging of hepatic nodules

**Fig. 3:**
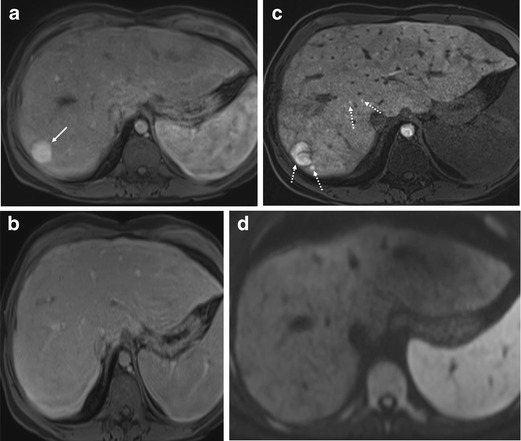
Focal nodular hyperplasia-like nodules in post-Fontan patients. **a**-**d** A 21-year-old woman with previous double inlet left ventricle. **a** There is an approximately 2.3 cm homogeneous hypervascular nodule in segment 7, periphery of the liver (*arrow*) on the axial arterial phase post contrast T1-weighted MR image. **b** The lesion showed iso-signal intensity with no hypointensity on the axial transitional phase post contrast T1-weighted MR image. **c** The lesion with a few other small foci (*dotted arrows*) showing increased contrast material uptake in the hepatobiliary phase T1-weighted MR image with gadoxetate disodium. **d** Single-shot echo-planar diffusion-weighted image (*b* = 1,000 s/mm^2^) demonstrates no diffusion restriction in the corresponding lesion

Large regenerative hepatic nodules are commonly seen in adult patients with FALD. The pathophysiology of the development of the nodules is not fully elucidated, although it has been hypothesised that central venous hypertension transmitted directly to the hepatic peri-venular areas diminishes portal blood flow and promotes arterialisation [44–46]. The prevalence of the nodules in adult patients with Fontan circulation ranges from 20 to 30% [11, 44, 46]. The nodules have been named both large regenerative nodules and focal nodular hyperplasia (FNH)-like nodules in the literature and can be found in patients with other causes of hepatic venous outflow obstruction such as Budd-Chiari syndrome or right heart failure [38, 47–49]. The nomenclature of “FNH-like nodule” comes from imaging features [38, 46, 50] of hyper-enhancement in the arterial phase and iso-enhancement in the delayed phase (Fig. 3a-c) and from histopathological features [44] of small islands of hepatic parenchyma separated by bands of arterialised vascular fibrous septa with a pseudo-capsule, similar to FNH.

**Fig. 4:**
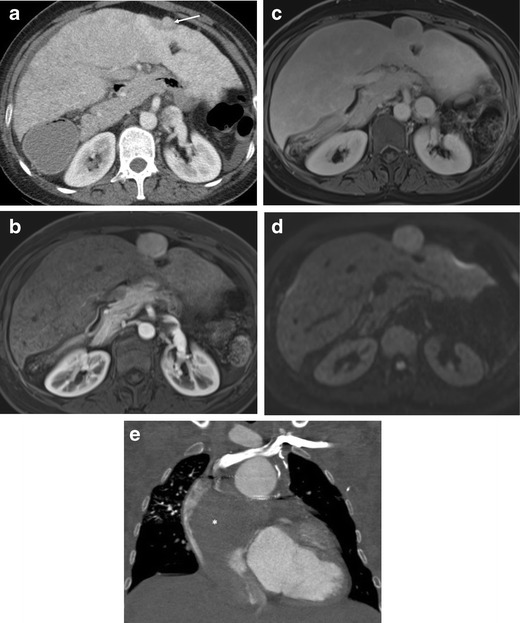
Hepatocellular carcinoma with extensive tumour thrombus in a post-Fontan patient. A 38-year-old woman with previous right atrial isomerism and pulmonary atresia. **a** Axial CT image in the portal venous phase demonstrates a 1.1-cm iso-attenuated nodule causing surface contour abnormality in segment 2 of the liver (*arrow*). This lesion was initially reported as regenerative nodule. **b**-**d** On 6-month follow-up MRI, the lesion increased to 3.4 cm, showing arterial enhancement (**b**), washout in 3-min delay (**c**), and strong diffusion restriction in single-shot echo-planar diffusion-weighted image (*b* = 1,000 s/mm^2^) (**d**). Biopsy confirmed the lesion as HCC and the lesion was successfully treated with radiofrequency ablation (RFA). **e** Local recurrence of segment II in the dome HCC directly invaded the inferior vena cava (not shown) and subsequently an extensive tumour thrombus developed and filled the inferior vena cava, the right atrium (*asterisks*) and extended to the left main pulmonary artery

Unfortunately, HCC can develop in adult patients with FALD [25, 26, 32, 46, 51] (Fig. 4) although the incidence of HCC is much lower than that of FNH-like nodules. Routine surveillance in FALD for HCC has been suggested, but there has been no consensus regarding the frequency of surveillance, the type of imaging modalities and the time interval after Fontan procedure. Progression from a dysplastic nodule to HCC is a potential stepwise pathway in liver cirrhosis imposed by Fontan circulation. However, malignant potential of FNH-like nodules is uncertain and has been controversial [18, 24, 44, 46]. Alleged benign large regenerative nodules in the chronic congested liver often mimic HCC radiologically and may render a correct diagnosis difficult [38]. Interval increase in size, washout in the portal venous phase, mosaic architecture, and elevated alpha-fetoprotein may be associated with HCC [22, 38].

FNH-like nodules in FALD are often small and multiple, and show a predilection for the right lobe in the periphery (mostly within 2 cm of the liver surface) [20]. On CEUS, FNH-like nodules in FALD show enhancement features similar to FNH; hyper-enhancement in the arterial phase, centrifugal enhancement, central stellate vasculature and sustained enhancement without washout (Supplementary material 1). MRI using liver-specific contrast agent is useful for characterising FNH-like nodules by demonstrating iso- or hyper-enhancement in the hepatobiliary phase. The FNH-like nodules may show central hypointensity in the hepatobiliary phase representing a central scar (Fig. 3c).

HCC typically shows [25, 32, 51–57] arterial-phase hyper-enhancement followed by late and mild washout on dynamic contrast-enhanced imaging. CEUS is potentially useful for indeterminate hepatic nodules in FALD on CT or MRI, especially for the patients who are contraindicated for contrast-enhanced CT or MRI. Suspicion of HCC is raised upon large size, interval change in size or echogenicity, mass-like appearance or nodules causing contour abnormality on the liver surface (Fig. 4a). However, it is important to note that the FNH-like nodules can infrequently demonstrate washout in the delayed phase, mimicking HCC [58, 59] (Supplementary material 1). The reported incidence of delayed-phase washout in FNH-like nodules is approximately 10%, in the setting of cardiac cirrhosis secondary to constrictive pericarditis and alcoholic cirrhosis. The pathophysiology for washout of FNH-like nodules is not clear. Wells et al. [22] speculated that the washout may not be related to an abnormality of the nodule itself, but a reflection of the background parenchymal contrast retention due to parenchymal congestion and fibrosis.

MRI using liver-specific contrast agent may be useful for the differentiation between FNH-like nodules and HCC, as HCC mostly show hypointensity in the hepatobiliary phase. DWI may be also useful in differentiating between HCC and FNH-like nodules. In our experience of a single case of HCC in FALD, HCC showed strong restricted diffusion, whereas FNH-like nodules did not show diffusion restriction, compatible with the study by Wells et al. [22] (Figs. 3d and 4d). The authors suggested other ancillary findings favouring benign FNH-like nodules, including hypointensity on T2-weighted images.

### Imaging of vascular abnormalities

On Doppler US, normal hepatic vein shows a triphasic waveform, consisting of a hepatopetal (coming towards the liver) phase occurring during atrial systole and two hepatofugal phases related to atrial and ventricular diastole. Loss of the hepatopetal phase is typically considered to represent increased stiffness of the liver such as in hepatic fibrosis, steatohepatitis and cirrhosis [60–62].

**Fig. 5:**
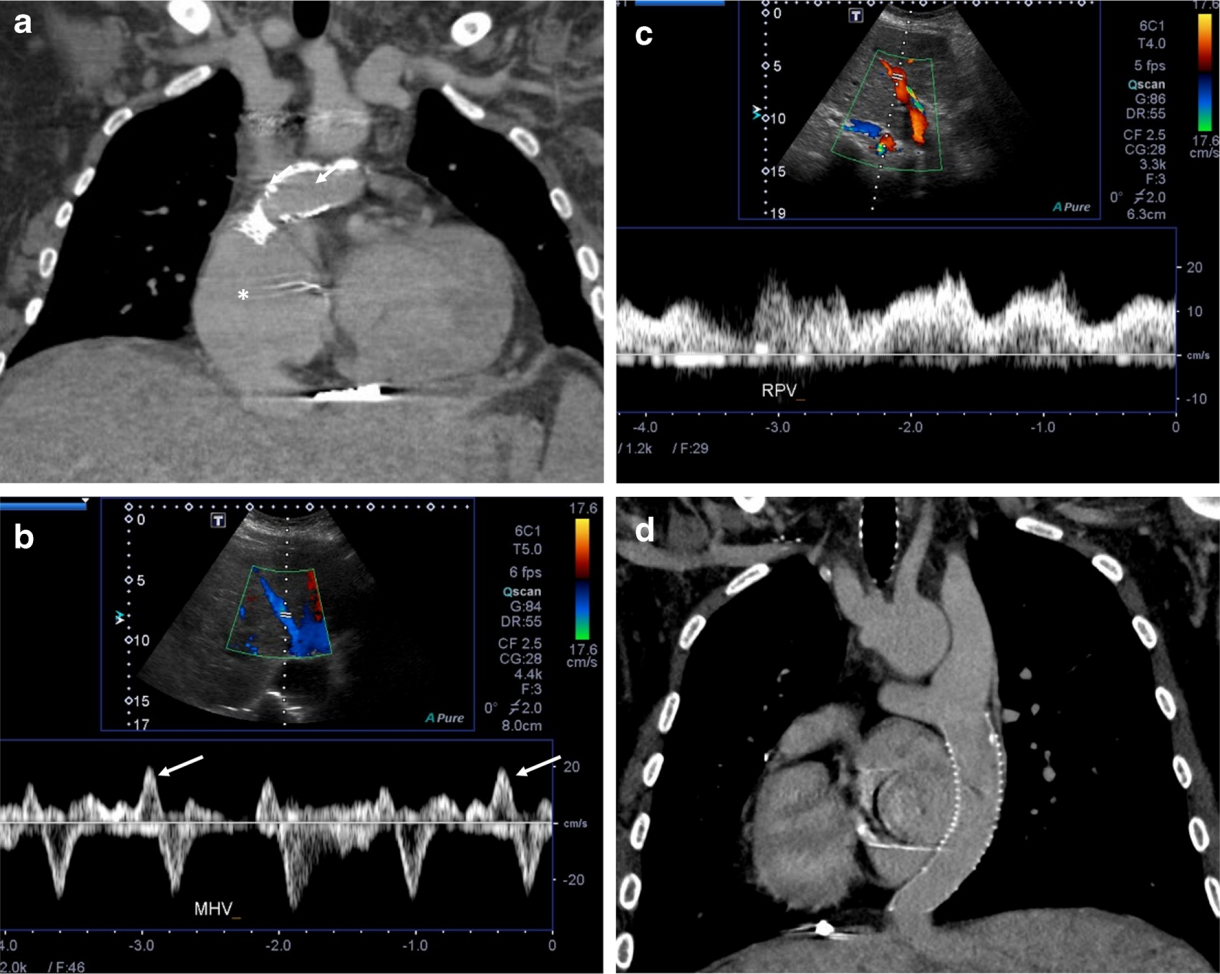

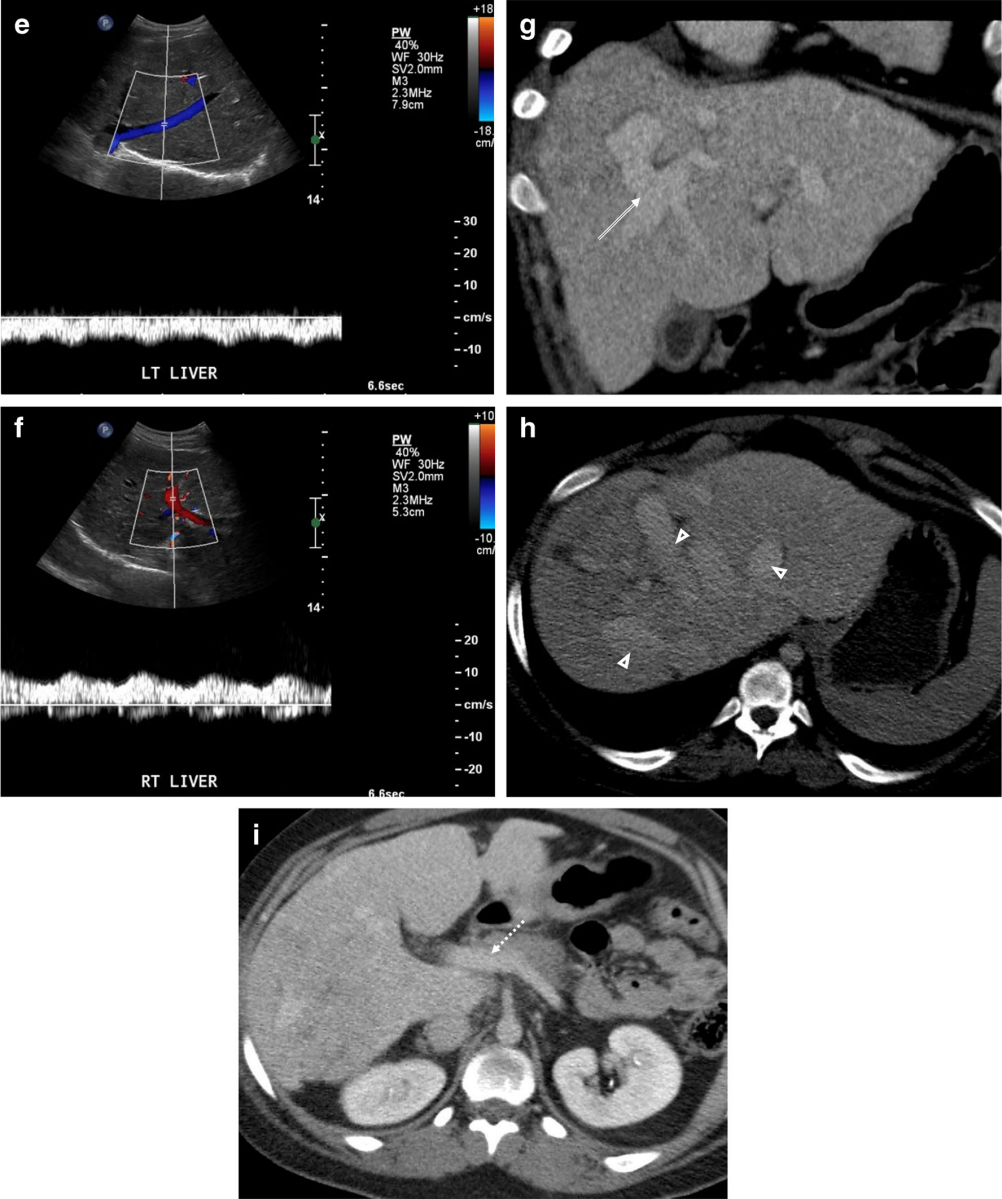
Vessel evaluation in post-Fontan patients. **a**-**c** A 31-year-old woman with previous pulmonary atresia with intact ventricular septum. **a** Coronal CT shows indirect diversion of both SVC and IVC to pulmonary arteries via the right atrium (*asterisk*), corresponding with atriopulmonary anastomosis. **b**, **c** Spectral Doppler US examination. **b** Middle hepatic vein shows preservation of hepatopetal blood flow (*arrows*). **c** Right portal vein shows heterogeneously coarse hepatopetal flow. **d**-**f** A 21-year-old woman with previous left atrial isomerism with pulmonary atresia. **d** Coronal CT shows direct diversion of both the SVC and IVC to pulmonary arteries, corresponding with total cavopulmonary anastomosis. Situs inversus is also noted. **e**, **f** Spectral Doppler US examination. **e** Left-side hepatic vein demonstrates disappearance of hepatopetal blood flow. **f** Right-side portal vein demonstrates relatively homogeneous hepatopetal flow. **g**-**i** A 33-year-old woman with previous hypoplastic right ventricle with tricuspid atresia. Coronal (**g**) and axial (**h**, **i**) CT images in the portal venous phases demonstrate intrahepatic venous-venous collateral formation (*double-lined arrow* in **g**), enlarged hepatic veins (*arrowheads* in **h**), and small main portal vein (*dotted arrow* in **i**)

Fontan operation inevitably alters hepatic venous waveform on Doppler US. The pattern of hepatopetal phase in hepatic vein differs between patients with total cavopulmonary anastomosis (including both lateral tunnels and extracardiac conduits) and with atriopulmonary connection [63–66] (Fig. 5a–e). Hepatopetal phase is preserved in atriopulmonary connection, which reflects the exclusion of atrial contribution to the venous circulation, whereas flow reversal is only visualised during early expiration in total cavopulmonary anastomosis. Also, similar to congestive heart failure, hepatic veins and IVC are dilated with abnormally increased pulsatility of the hepatic vein regardless of the anastomosis technique [38, 61, 67].

Portal venous waveform on Doppler US normally shows slightly phasic hepatopetal flow with mildly decreased flow during inspiration due to transient compression of the compliant liver parenchyma and easily collapsible portal venules and hepatic sinusoids [61, 68]. In patients with Fontan circulation, the portal veins invariably show hepatopetal flow with variable phasicity, but the flow velocity is lower than in the normal population [69, 70]. While the dilatation of the IVC and hepatic veins is frequently seen in post-Fontan patients, the diameter of the main portal vein and intrahepatic portal veins is usually small, possibly due to reduction of portal perfusion secondary to increased sinusoidal pressure with venous stasis [39] (Fig. 5). Bland portal vein thrombosis seems to be rare in post-Fontan patients with no reported cases compared to Budd-Chiari syndrome, where portal vein thrombosis occurs up to 18% [71, 72]. Hepatofugal flow of the portal vein, which can be found in severe portal hypertension or Budd-Chiari syndrome with large extrahepatic portosystemic collaterals, has not been reported in post-Fontan patients [63, 66, 70, 73–75] . It can be explained by relatively high systemic venous pressure in Fontan circulation, preventing the development of large extrahepatic portosystemic collaterals.

On the arterial-phase CT with contrast injection through the upper extremity veins, dense opacification in the dependent portion of the IVC and hepatic veins is occasionally seen in post-Fontan patients. This is likely due to passive retrograde flow of the contrast material which is heavier than blood in the absence of effective right heart function (Supplementary material 2). This phenomenon might be different from the opacification of IVC and all hepatic veins in patients with right heart failure [38, 76, 77], which is the result of reflux of contrast media into the inferior vena cava during right atrial contraction or tricuspid regurgitation.

## Extrahepatic findings

### Signs of portal hypertension

Extrahepatic complications of portal hypertension secondary to liver cirrhosis such as splenomegaly and ascites are commonly seen in adult Fontan patients. Compared to liver cirrhosis secondary to hepatitis, extrahepatic portosystemic shunts are uncommon or small in size in FALD, even in a decompensated state, likely due to the high systemic venous pressure in Fontan circulation and relatively low pressure gradient between the portal and systemic veins to promote the formation of large portosystemic collaterals [38] (Supplementary material 3).

### Protein losing enteropathy

Protein losing enteropathy (PLE) is characterised by enteric loss of proteins including albumin, immunoglobulins and clotting factors. Patients with PLE present with peripheral oedema, ascites, diarrhoea, weight loss and malabsorption. The reported incidence of PLE in Fontan patients ranges from 3 to 18%, with a reported mortality of 50% within 5 years after initial diagnosis [78–80]. Elevated systemic venous pressure and mesenteric vasoconstriction, which is the natural reaction to decreased cardiac output, might be the cause of PLE. Additionally, lymphatic dilatation, stasis and leakage, and inflammatory reaction secondary to elevated systemic venous pressure, further contribute to the development of PLE [19, 80, 81].

Early clinical diagnosis of PLE is often difficult because the patients may have a long asymptomatic period [80–82]. As multiple intensive therapeutic approaches enable an improved survival rate of up to 88% at 5 years [83], early recognition of excessive enteric loss of protein is important. Suggestive imaging findings including ascites, diffuse bowel wall thickening, and severe mesenteric oedema along the root of the mesentery on CT or MRI, should prompt a clinical evaluation to rule out PLE [13].

### Pheochromocytoma and paraganglioma

**Fig. 6:**
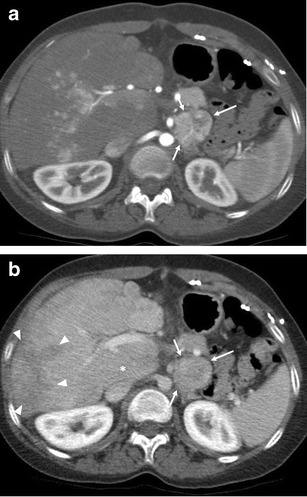
Pheochromocytoma in a post-Fontan patient. A 24-year-old woman with previous hypoplastic right ventricle with dysplastic tricuspid valve. Axial CT images in the arterial (**a**) and portal venous (**b**) phases demonstrate a 4.5-cm heterogeneous enhancing mass originating from left adrenal gland (*arrows*). The patient underwent an operation and the lesion was confirmed as pheochromocytoma. Note numerous arterial enhancing foci with no delayed washout scattered in the liver in the arterial phase and heterogeneous poor parenchymal enhancement in the periphery of the liver in the portal venous phase (*arrowheads*). Caudate lobe is hypertrophied with relative homogeneous enhancement (*asterisk*)

Pheochromocytoma and paraganglioma are neuroendocrine tumours arising from neural crest-derived cells or organs in the adrenal gland and along the central sympathetic and parasympathetic chains, respectively. There have been several anecdotal reports of the development of pheochromocytoma or paraganglioma in patients with congenital cyanotic heart disease after corrective or palliative cardiac surgery, including the Fontan procedure [84–90]. Suggested pathophysiology is a stimulation of catecholamine overproduction and chronic endocrine hyperactivity resulting from the hypoxic state of congenital heart disease [91]. The tumours may cause palpitations, progressive arrhythmia, fatigue and malaise. Pheochromocytoma or paraganglioma are typically seen as heterogeneously and avidly hyper-enhancing masses with areas of necrosis and cystic component [92, 93] (Fig. 6).

## Conclusions

Fontan procedures, designed to treat patients with functional single ventricles, have markedly improved the patient’s survival into adulthood. The physiology of the Fontan circuit inevitably increases systemic venous pressure, which may lead to multi-system organ failure in the long-term follow-up. FALD is most common among post-Fontan abdominal complications and can progress to liver cirrhosis with signs of portal hypertension. FNH-like nodules frequently develop in FALD as small multiple nodules with arterial-phase hyper-enhancement and show imaging findings similar to FNH. Imaging surveillance is often performed to monitor the progression of FALD to liver cirrhosis and to detect HCC, which infrequently develops in FALD. Extrahepatic abdominal abnormalities in post-Fontan patients include signs of portal hypertension such as ascites, splenomegaly and varices, protein losing enteropathy, and pheochromocytoma/paraganglioma. Given that these abdominal abnormalities are critical for patient management, it is important to be familiar with the typical imaging findings of the abnormalities on cross-sectional imaging.

## Electronic supplementary material

ESM 1 (DOCX 7073 kb)
