# Supplementary material for: Abdominal imaging findings in adult patients with Fontan circulation
Source: Insights Imaging. 2018 Apr 5;9(3):357–67. doi: 10.1007/s13244-018-0609-2 (PMC5991003; doi:10.1007/s13244-018-0609-2)
Supplement: Supplementary file 1 — (DOCX 7073 kb) [file 13244_2018_609_MOESM1_ESM.docx]

**Supplementary material**

**A B**

**
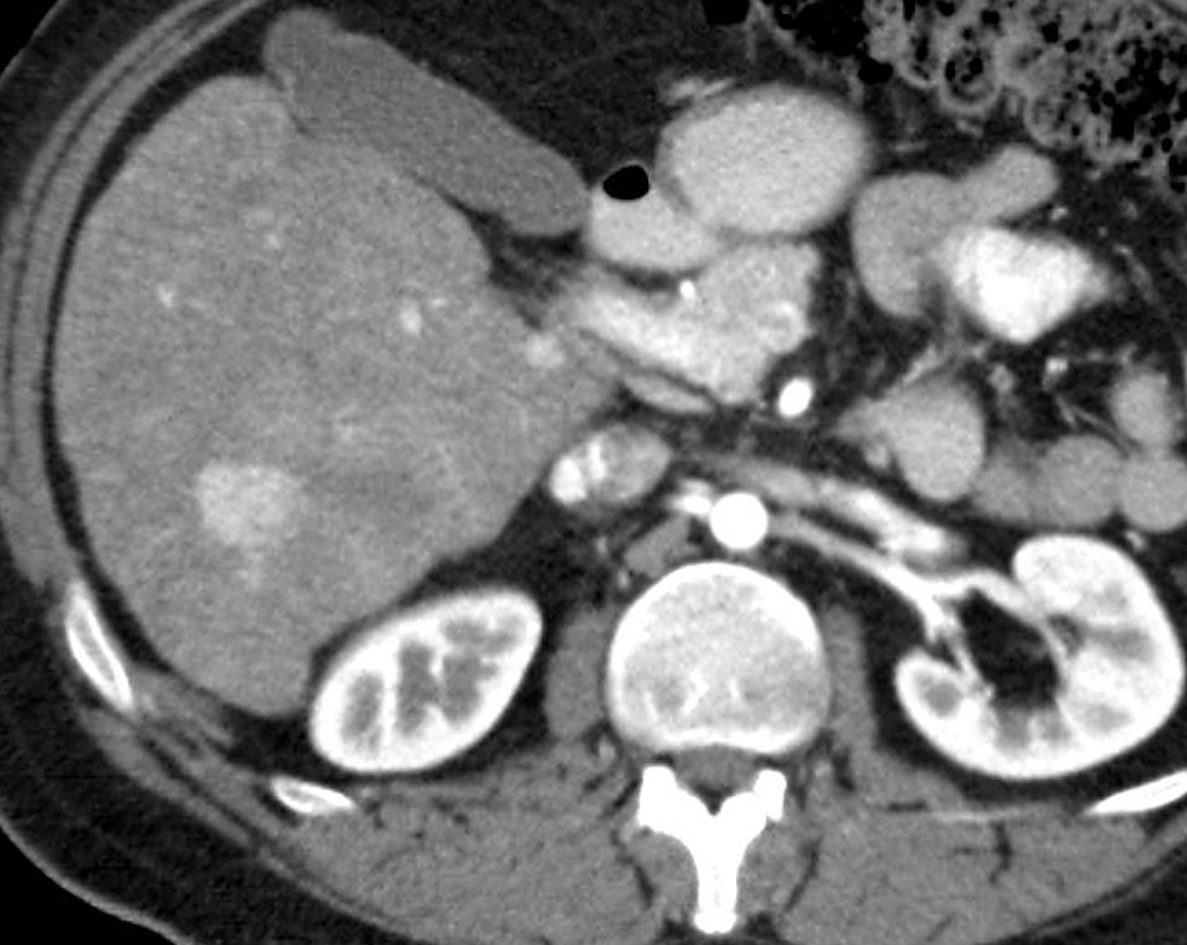

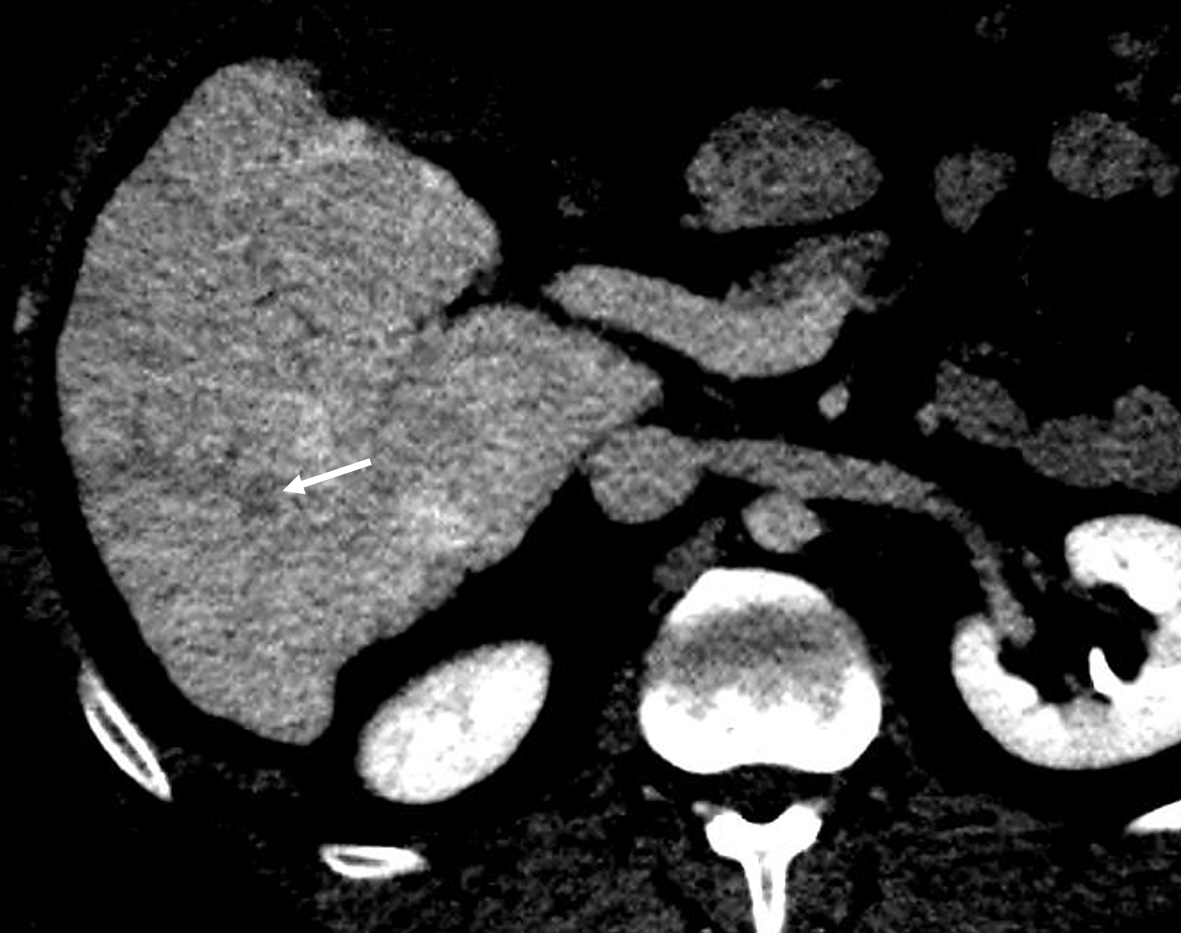
**

**C D**

**
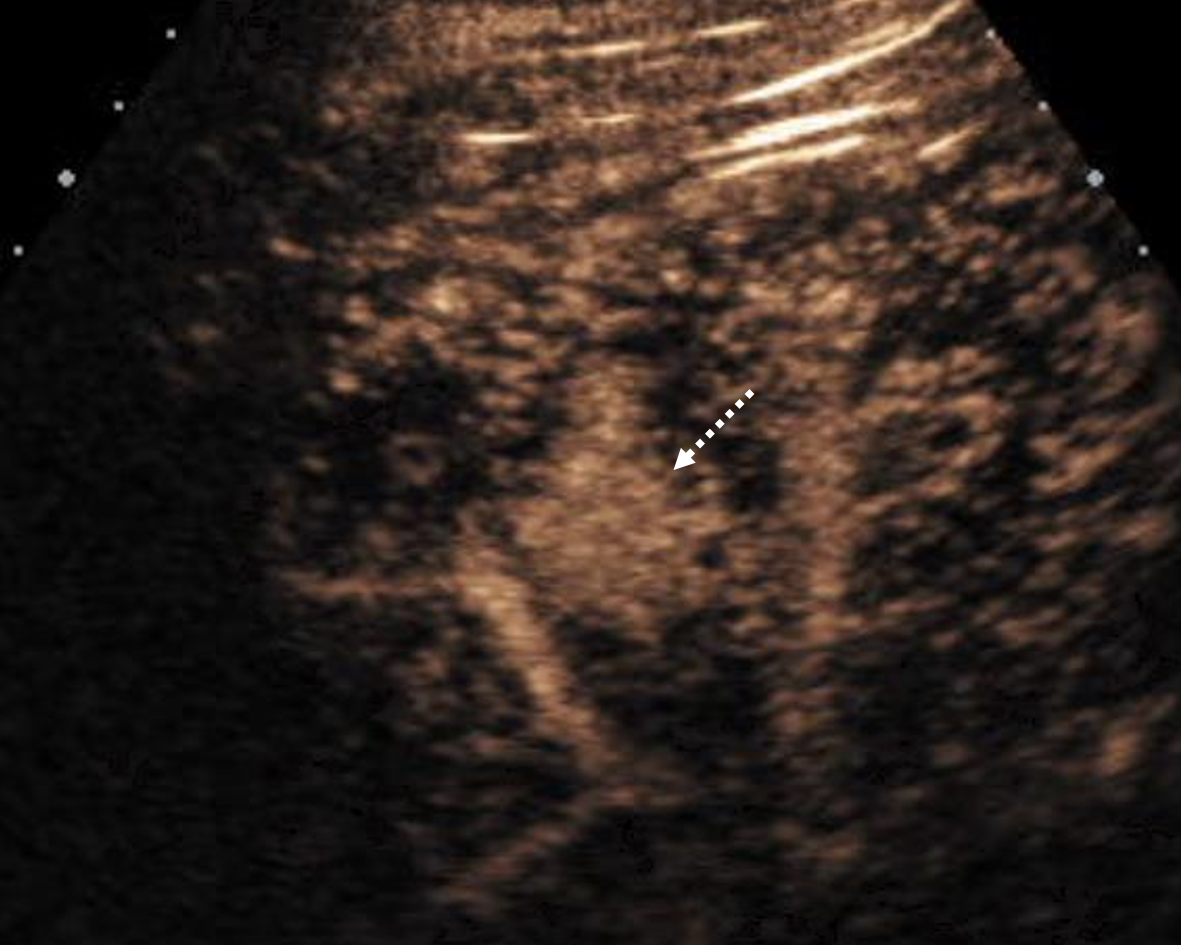

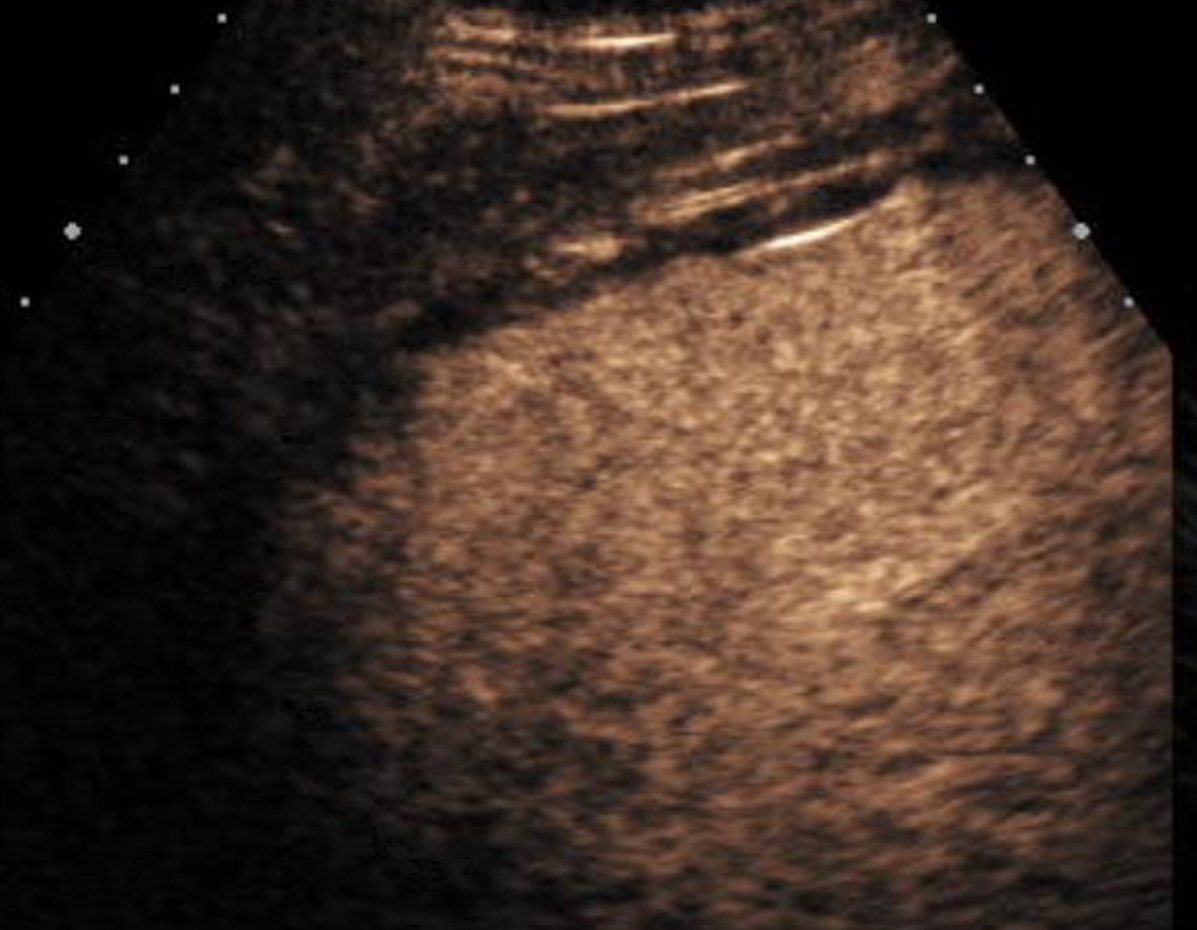
**

**Supplementary 1. Atypical focal nodular hyperplasia-like nodule in CT and contrast-enhanced US in a post-Fontan patient.**

32 year old female patient with previous tricuspid atresia.

(A, B) Axial CT images in the arterial (A) and portal venous (B) phases demonstrate 2.4cm arterial enhancing nodule in S6 of the liver with focal area of washout in portal venous phase (arrow in (B)).

(C, D) Consecutive contrast enhanced ultrasound image obtained at 30 seconds (C) and 90 seconds (D) show arterial enhancement (dotted arrow in (C)) with no washout, favouring focal nodular hyperplasia-like nodule rather than hepatocellular carcinoma. Biopsy confirmed the lesion as focal nodular hyperplasia-like nodule.


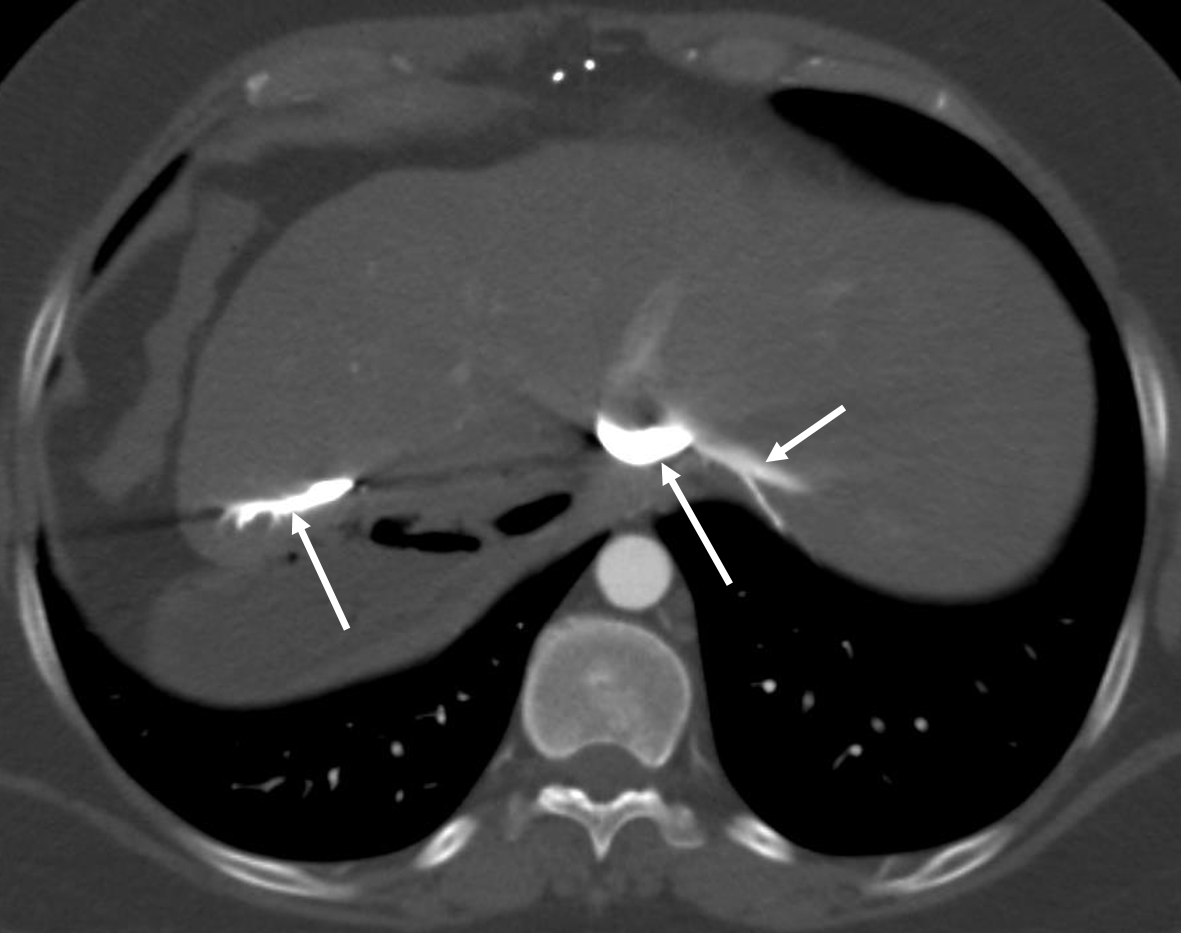


**Supplementary 2. Passive opacification of inferior vena cava and hepatic veins during early arterial phase in CT in a post-Fontan patient.**

22 year old female patient with previous right atrial isomerism and pulmonary atresia.

Axial CT image in the early-arterial phase demonstrate iodinated contrast media directly transmits to inferior vena cava and passively flows to both hepatic vein along gravity (arrows).

**A B**


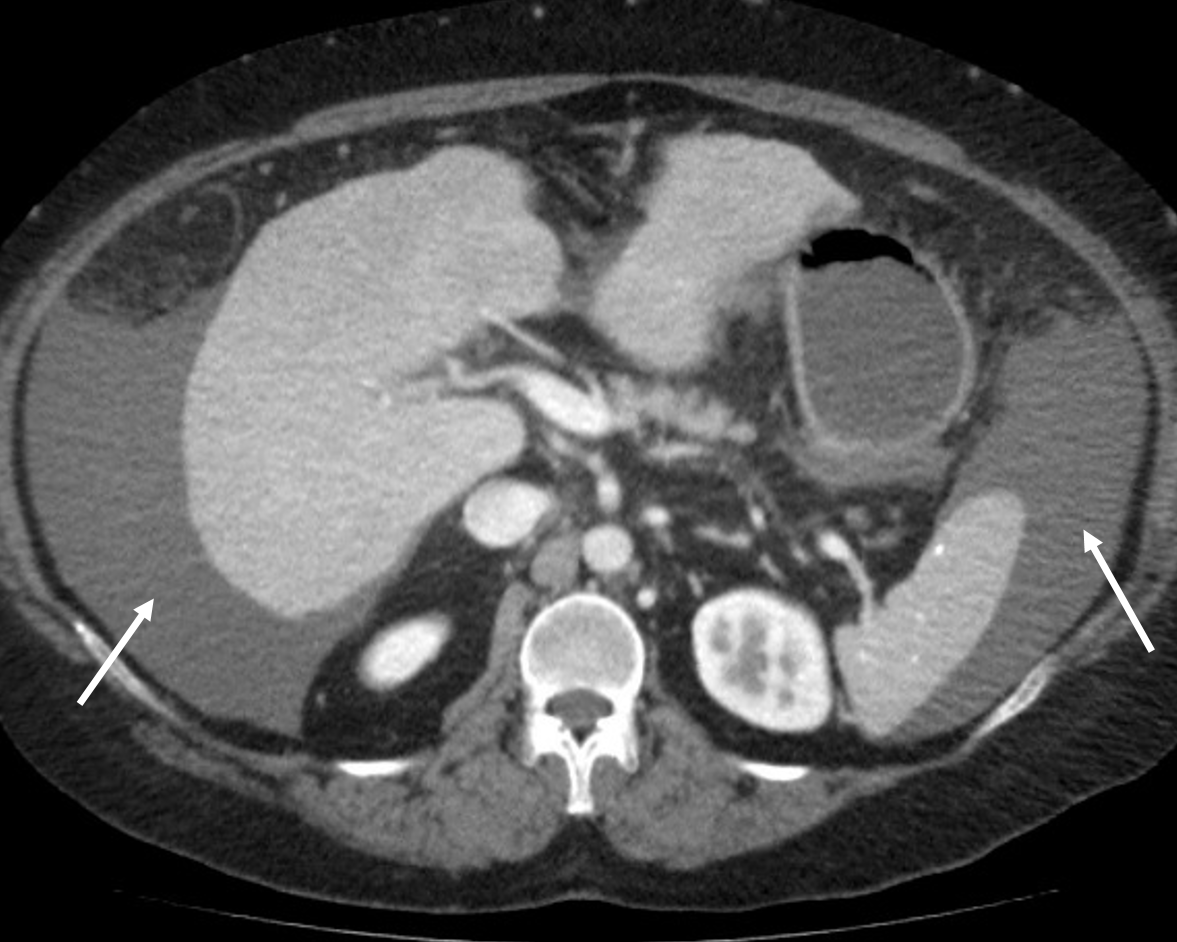

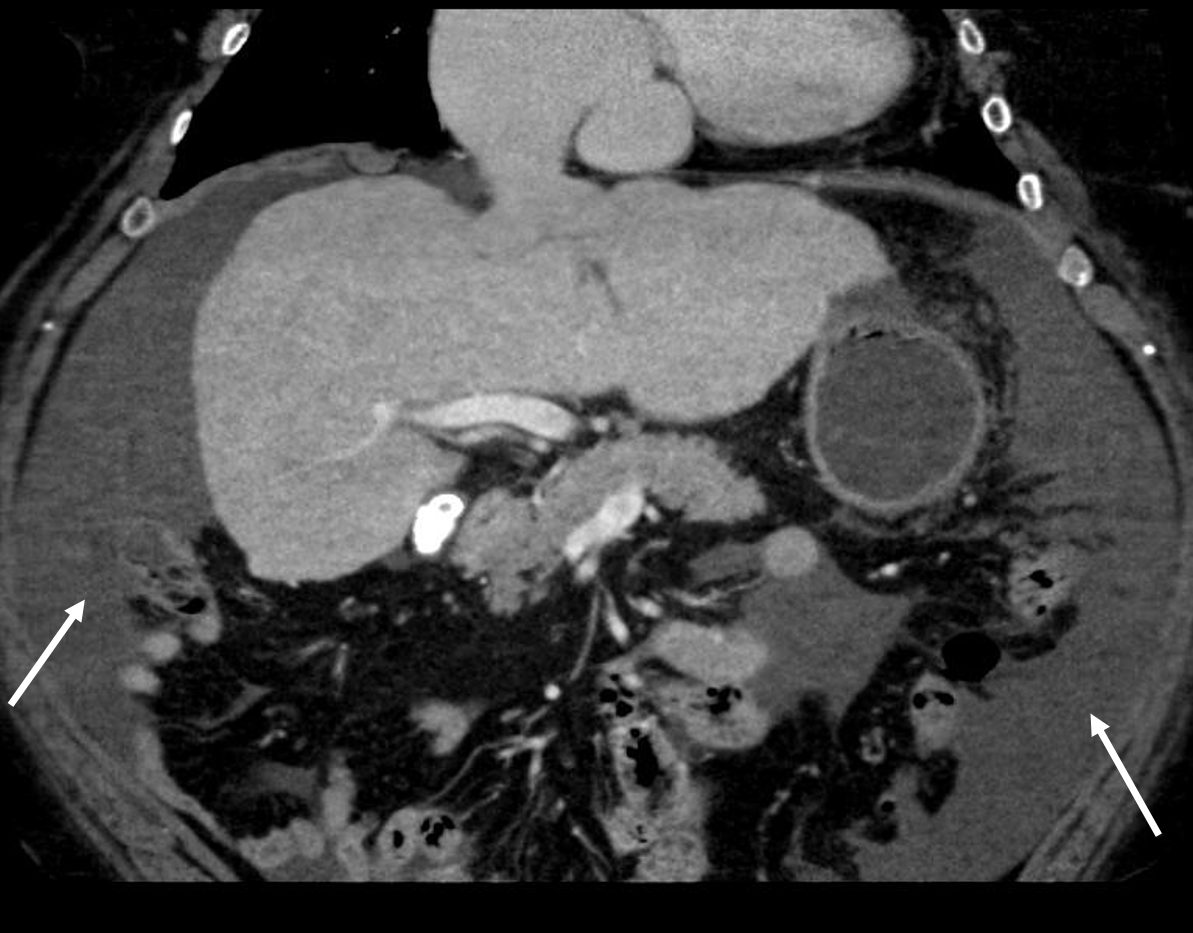


**Supplementary 3. Decompensated liver cirrhosis in CT in a post-Fontan patient.**

38 year old female patient with previous hypoplastic right ventricle syndrome. Axial (A) and coronal (B) CT images in the portal venous phases show heterogeneous enhancement with lobulating hepatic contour and large amount of ascites (arrows). Despite the findings of decompensated liver cirrhosis, no evidence of portosystemic shunting is visualised.
